# Loud Noise Exposure Produces DNA, Neurotransmitter and Morphological Damage within Specific Brain Areas

**DOI:** 10.3389/fnana.2017.00049

**Published:** 2017-06-26

**Authors:** Giada Frenzilli, Larisa Ryskalin, Michela Ferrucci, Emanuela Cantafora, Silvia Chelazzi, Filippo S. Giorgi, Paola Lenzi, Vittoria Scarcelli, Alessandro Frati, Francesca Biagioni, Stefano Gambardella, Alessandra Falleni, Francesco Fornai

**Affiliations:** ^1^Department of Clinical and Experimental Medicine, University of PisaPisa, Italy; ^2^Department of Translational Research and New Technologies in Medicine and Surgery, University of PisaPisa, Italy; ^3^Istituto di Ricovero e Cura a Carattere Scientifico IRCCS NeuromedIsernia, Italy

**Keywords:** hippocampus, striatum, cerebellum, cell damage, loud noise, rat

## Abstract

Exposure to loud noise is a major environmental threat to public health. Loud noise exposure, apart from affecting the inner ear, is deleterious for cardiovascular, endocrine and nervous systems and it is associated with neuropsychiatric disorders. In this study we investigated DNA, neurotransmitters and immune-histochemical alterations induced by exposure to loud noise in three major brain areas (cerebellum, hippocampus, striatum) of Wistar rats. Rats were exposed to loud noise (100 dBA) for 12 h. The effects of noise on DNA integrity in all three brain areas were evaluated by using Comet assay. In parallel studies, brain monoamine levels and morphology of nigrostriatal pathways, hippocampus and cerebellum were analyzed at different time intervals (24 h and 7 days) after noise exposure. Loud noise produced a sudden increase in DNA damage in all the brain areas under investigation. Monoamine levels detected at 7 days following exposure were differently affected depending on the specific brain area. Namely, striatal but not hippocampal dopamine (DA) significantly decreased, whereas hippocampal and cerebellar noradrenaline (NA) was significantly reduced. This is in line with pathological findings within striatum and hippocampus consisting of a decrease in striatal tyrosine hydroxylase (TH) combined with increased Bax and glial fibrillary acidic protein (GFAP). Loud noise exposure lasting 12 h causes immediate DNA, and long-lasting neurotransmitter and immune-histochemical alterations within specific brain areas of the rat. These alterations may suggest an anatomical and functional link to explain the neurobiology of diseases which prevail in human subjects exposed to environmental noise.

## Introduction

Noise pollution has become a severe issue in public health. During daily life, people are exposed to hazardous noise levels produced by a variety of sources such as work environment, urban traffic, household appliances, loud music, etc. (Kawecka-Jaszcz, 1991; Lang et al., 1992). The World Health Organization estimated that roughly 20% of Europeans are exposed to loud noise generated by urban traffic exceeding 65 dBA, which represents the safety threshold (Berglund et al., 1999). Approximately 40% is exposed to noise levels between 55 dBA and 65 dBA (below the safety threshold), which when reiterated over time, might still contribute to the onset of a number of disorders (Berglund et al., 1999).

For instance, it has been estimated that the burden of disease from environmental noise leads to 61,000 disability-adjusted life-years lost (DALYs) due to hypertension-related ischemic heart disease and related heart disorders; 45,000 DALYs derive from cognitive impairment which occurs even in children and young people. 903,000 DALYs are related to sleep disorders specifically for people living in towns owing more than 50,000 inhabitants. 22,000 DALYs are due to tinnitus. The DALYs attributed to noise in Western European countries were more than those attributed to lead (100–900), ozone (30–140) and dioxin (200–600) (WHO (World Health Organization), 2011).

The amount of noise required to produce these disabilities may vary. In fact, epidemiological studies indicate that, even exposure to a noise intensity of <60 dB (below the conventional safety threshold) may trigger depressive symptoms (Orban et al., 2016). Exposure to >60 dB induced by road and railway traffics is associated with pathological adiposity (Christensen et al., 2016). We have shown that exposure to loud noise (100 dBA) induces ultrastructural alterations in the rat myocardium which are accompanied by DNA damage (Lenzi et al., 2003). Similarly, a loss of DNA integrity was also assessed in the adrenal gland of loud noise-exposed rats (Frenzilli et al., 2004). It is very likely that a damage to these peripheral organs derives from altered sympathetic innervation, which in turn depends on altered brain circuitries (Lenzi et al., 2003; Christensen et al., 2016; Hjortebjerg et al., 2016). In fact, loud noise exposure is expected to affect internal organs via its natural gateway, the inner ear, thus altering brain areas governing neuroendocrine functions.

For instance, loud noise induces neuropsychiatric effects such as anxiety, emotional stress and psychiatric disorders (Rabat, 2007), which in turn are expected to alter the hypothalamic pituitary axis (Lenzi et al., 2003; Christensen et al., 2016). It is remarkable that residential road traffic in early childhood produces dose-dependently psychiatric symptoms such as hyperactivity and impaired attention (Hjortebjerg et al., 2016), which are reminiscent of attention deficit hyperactivity disorder (ADHD). Thus, loud noise configures as a powerful stressor which may alter the central core of the brain which regulates both autonomic and archaic behavioral responses. The sleep-waking cycle is markedly altered by loud noise (WHO (World Health Organization), 2011), which again, suggests an altered activity of those archaic brain areas such as the reticular formation, which convey stressful stimuli throughout the brain.

Most studies on the effects of loud noise in the central nervous system (CNS) focused on the hippocampus only (Busceti et al., 2015), which indeed is a direct target of the reticular formation (Fornai et al., 2011; Ruffoli et al., 2011). However, this is due more to serendipity triggered by the inspiring role of the hippocampus than to an exhaustive whole brain analysis. In fact, data from other brain areas are still missing or, when present, they are scattered. For instance, loud noise is known to produce cerebellum-dependent behavioral alterations (Uran et al., 2010) while altered monoamine innervation is produced by loud noise in the striatum (Tsai et al., 2005; Hu et al., 2014). Again, in our previous studies we found that in mice, a brief exposure to loud noise when associated to a sub-threshold dose of 3,4-methylendioxymethamphetamine (MDMA, ecstasy) produces a loss of striatal dopamine (DA) levels and nigrostriatal innervation. In summary, no study compared different brain areas using various experimental procedures following loud noise exposure. Therefore in the present study, we described cerebellar and striatal alterations along with hippocampal changes by using different approaches ranging from analysis of DNA integrity to neurotransmitter assay and pathological studies in rats exposed to loud noise. In particular, due to a massive involvement of the hippocampus in the effects of loud noise, we focused on the potential noise-induced hippocampal alterations. It is reported that loud noise exposure induces hippocampal damage such as apoptosis (Säljö et al., 2002; Cui et al., 2013; Kim et al., 2013), and tau-phosphorylation (Cui et al., 2012, 2013; Cheng et al., 2016).

In this experimental study we chose the noise level (100 dBA) and exposure duration (12 h) which are similar to that previously shown to be effective in altering heart and adrenal gland (Lenzi et al., 2003; Frenzilli et al., 2004). Incidentally, this corresponds to noise levels which are comparable with those occurring in specific contexts (i.e., rave parties, discos, aircraft work place) during daily life.

By combining biochemistry, immunohistochemistry and an assay of DNA integrity we were able to analyze the concomitancy or the time sequence of noise-induced focal brain alterations, which are likely to be connected in a chain of molecular events and through anatomical pathways encompassing various brain regions. These results shed new light on the symptoms associated with loud noise exposure.

## Materials and Methods

### Animals

Male Wistar rats, weighing 200–250 g (Harlan Labs, San Pietro al Natisone, Italy) were used for the experiments. Animals were housed in the animal facility, they were fed *ad libitum* and they were kept under closely controlled environmental conditions (12 h light/dark cycle, lights on between 07:00 h and 19:00 h; room temperature 21°C). All *in vivo* treatments were carried out in 2003, when we described the deleterious effects of loud noise on the pituitary-adrenal axis. At that time the experiments were carried out in compliance with norms and guidelines formulated by the European Council (86/609/EEC) which represented the gold standard reference for the use and care of laboratory animals. All possible efforts were made to reduce animal suffering and we reduced the number of animals used while granting statistical power.

### Loud Noise Exposure

Seven days before noise exposure, rats were housed individually in the experimental cage, to avoid any possible cage- and isolation-induced stressful effect. In fact, during noise exposure rats were housed one per cage to avoid that they might shield each other against loud noise. Therefore, noise-exposed rats (*n* = 22) were individually placed for 12 h in cages close to loud speakers (15 W) mounted, 40 cm apart, on opposite sides of the cage and activated by a white-noise generator (0–26 kHz). The noise level was set at 100 dBA (Frenzilli et al., 2004) and was uniform inside the cage, as monitored with a sound meter (Quest Electronics 215).

Control rats (*n* = 22) were individually placed in the same cage size for 12 h, but not being exposed to noise.

### Experimental Procedures

Animals were killed immediately after noise stimulus (for Comet Assay, *n* = 4) or at 24 h (for light microscopy, *n* = 4) or 7 days later (for neurotransmitter analysis, *n* = 10, and, again, light microscopy, *n* = 4). Paralleled sacrifices of controls have been performed as well. The brain was immediately removed to dissect each brain areas (Fornai et al., 2004).

For immunohistochemistry at light microscopy, animals were anesthetized by i.p. injection with chloral hydrate (440 μL/100 g), they were thoracotomized and then, they were transcardially perfused by a fixing solution (about 300–350 mL/rat), preceded by a saline solution (about 200 mL/rat, anyway until liver appeared pale). The fixing solution consisted in 4% formaldehyde in a phosphate buffer solution (0.1 M, pH = 7.3, room temperature).

### Evaluation of DNA Damage

DNA integrity was evaluated by the use of alkaline single-cell gel electrophoresis or comet assay, according to Singh et al. (1988), with minor modifications (Fornai et al., 2004). Briefly, isolated cells are embedded in agarose on a microscope slide, lysed with detergent, and treated with high salt. Any breaks present in the DNA cause the supercoiling to relax locally, and negatively charged loops of DNA are then free to extend and migrate in the electric field toward the anode as a “comet tail”. After sacrifice, separate specimens from the three brain areas were washed in cold phosphate-buffered saline and then placed in 1 mL of chilled mincing solution (Ca^2+^, Mg^2+^-free Hank’s balanced salt solution, 20 mM Na_2_EDTA, 10% dimethyl sulfoxide, pH 7.5). The tissue was cut in small pieces by scissors.

**Figure 1:**
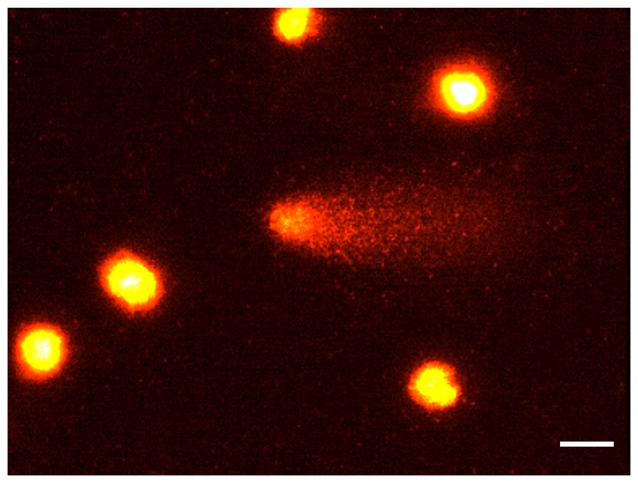
The comet assay. Rat brain nuclei processed for the Comet assay. Five nuclei with undamaged DNA and one nucleus with about 50% of DNA in the tail. Bar = 20 μm.

After 15 min, the supernatant was centrifuged for 10 min at 1000 rpm. The assessment of cell viability on individual cells was not possible in these conditions because during mincing and dissociation processes, cell membrane was disrupted (Singh et al., 1995). The pellet obtained was mixed with 75 μL of agarose (0.5% low–melting-point agarose (LMPA) prepared in Ca^2+^, Mg^2+^-free phosphate buffered saline) and layered on conventional slides, predipped in 1% normal–melting-point agarose (Klaude et al., 1996). Then, a third layer of 85 μL LMPA was added. Slides were immersed in ice-cold freshly prepared lysis solution (2.5 M NaCl, 100 mM Na_2_EDTA, 10 mM Tris-HCl, 1% Triton X-100, and10% dimethyl sulfoxide, pH 10) to lyse the cells and allow DNA unfolding. After 1 h at 4°C in the dark, slides were covered with an alkaline solution (1 mM Na_2_EDTA, 300 mM NaOH, pH >13) in a horizontal electrophoresis unit for 20 min to allow DNA unwinding and expression of alkali-labile sites. Slides successively underwent electrophoresis (25 V, 300 mA, 20 min) in an ice-cold bath. Next, slides were washed gently with a neutralization buffer (0.4 M Tris-HCl, pH 7.5) to remove alkali and detergents, dipped in 100% cold methanol, and dried. After drying, slides were stained with 100 μL of ethidium bromide (2 μL/mL). All the steps described above were conducted under yellow light or in the dark, to prevent nonspecific DNA damage. DNA migration is proportional to the level of DNA damage (Figure 1). For each experimental point four animals were used. For each brain area a total of at least 100 cells were scored and the mean calculated. Nuclei were observed under a fluorescence microscope (200×), and an image analyzer (Kinetic Imaging Ltd, Komet, version 4) was used.The effects of loud noise on DNA integrity in single cells dissociated from hippocampus, cerebellum and striatum was evaluated as the percentage of migrated DNA (Kumaravel and Jha, 2006), tail length (TL) and tail moment (TM) after electrophoresis. Comet data analysis was performed by three scorers blind to treatment.

### Diffusion Assay

**Figure 2:**
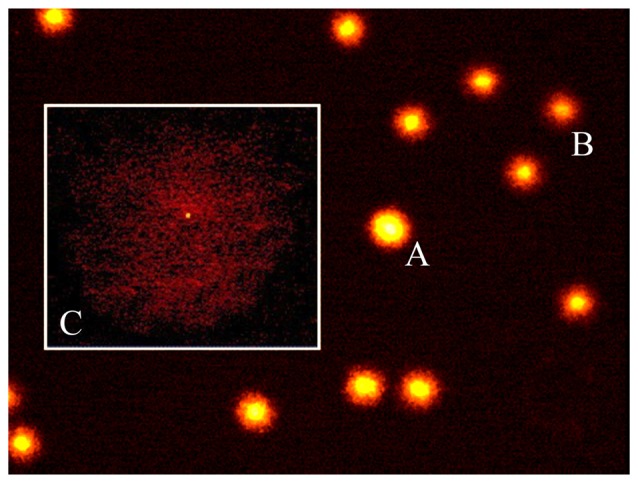
The diffusion assay. A photomicrograph of rat brain nuclei processed for the DNA diffusion assay. Non apoptotic cells at increasing degree of damaged DNA **(A,B)** and one apoptotic cell **(C)** are shown (magnification: 400×).

Because of the potential occurrence of very low molecular weight DNA which is produced by endogenous endonucleases when apoptosis occurs, we additionally carried out a diffusion assay since comet DNA may be lost from the gels under the typical electrophoretic conditions used (Tice et al., 2000). In non-electrophoretic conditions, apoptotic cells were identified by the presence of highly dispersed DNA giving rise to a characteristic halo around the nucleus (Singh, 2000). Cells with DNA damage only (neither necrotic nor apoptotic cells) are clearly defined, while apoptotic nuclei have a larger size with a projections of DNA all around (Figure 2). At least 100 cells per data point were scored and the percentage of apoptotic cells evaluated. Diffusion assay data analysis was performed by three scorers blind to treatment.

### Catecholamines Assay

**Figure 3:**
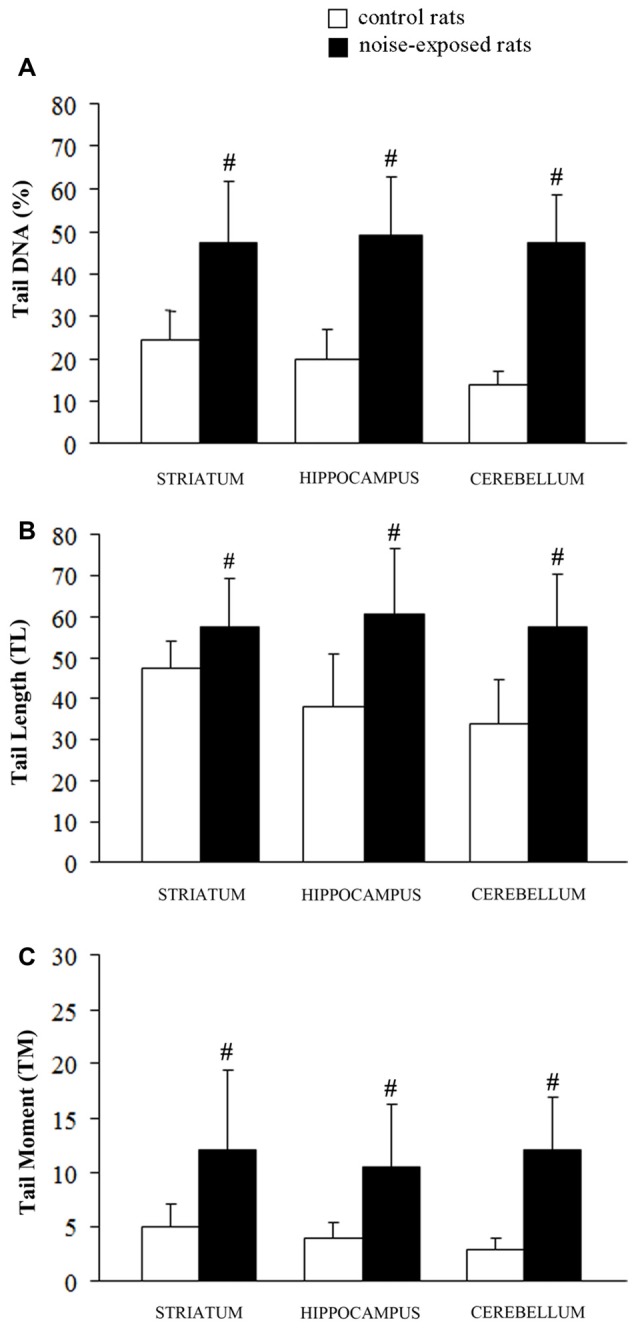
Loud noise exposure produces DNA fragmentation. DNA damage induced by loud noise exposure in three rat brain areas (striatum, hippocampus, cerebellum) soon after the cessation of the stimulus. Data are expressed as means values of **(A)** tail DNA (%), **(B)** tail length (TL) and **(C)** tail moment (TM). White column = control rats; black column = 12 h noise-exposed rats. Four animals per group were used and group summaries ± SD are presented. ^#^*p* < 0.001.

For catecholamine assay, the brains were rapidly removed and placed on ice-cold saline. Brain was first sagittally cut along the inter-emispheric scissure by using an ice-cold lancet, and then observed under stereomicroscope in order to correctly visualize the areas of interest. In particular, the rostral striatum was dissected out through the external wall of the lateral ventricle, while the ventral hippocampus was punched out, after a frontal cut carried out about 2.5 mm posterior to bregma, keeping the cerebral peduncles as a reference point. Finally, samples from cerebellar hemispheres were obtained by superficial dissection of the lateral organ at the level of lobus anterior. The cartoon of Figure 3 shows the procedure of striatal, hippocampal and cerebellar dissection. During the entire procedure, brains were placed on a Petri dish put on dry ice, in order to avoid potential bias due to the quick catecholamine oxidation.

Samples were homogenized in 600 μl of 0.1 N perchloric acid (PCA) containing diaminobenzylamine as the internal standard at the concentration of 10 pg/μl, using a sonicator. Fifty microliter of the homogenate were used to measure protein concentration. The samples were then centrifuged at 10,000 *g* for 5 min al 4°C. Twenty microliter of the supernatant were injected into a high-performance liquid chromatography (HPLC) apparatus gifted with an isocratic pump and equipped with a reverse-phase column (250 × 4.5 mm, C18, SGE) and two coulometric electrochemical detectors (Fornai et al., 1999). We decided to use the reducing as the revealing electrode to produce low level of electrical noise. The mobile phase consisted of a citrate-phosphate buffer (0.04 M citric acid, 0.06 M Na_2_HPO_4_ 2H_2_O) solution containing 0.1 mM ethylene diamine tetra-acetic acid (EDTA), 0.6 mM 1-heptanesulfonic acid sodium salt, and 10% methanol. The standard curve for each compound was calculated using a regression analysis as described in the “Statistical Analysis” Section.

### Light Microscopy

After dissection brains were post-fixed by immersion in the same fixing solution used for perfusion at 4°C for 24 h. Then brains were washed in distilled water, dehydrated in increasing alcohol solutions and plunged in xylene to be finally embedded in paraffin. Eight-micrometer thick sections were cut using a microtome and they were collected on poly-L-lysine (Sigma, St Louis, MO, USA)-coated slides. Sections from striatum, hippocampus and cerebellum were selected in order to check for anatomical integrity through hemeatoxylin-eosin and toluidine blue staining. In these sections we failed to find either necrotic areas or massive cell loss. This was also confirmed by using fluoro-Jade B staining although the optimal window to detect dying cells when using fluoro-Jade B produces a peak of signal between 24 h and 72 h after the noxious stimulus.

### Immunohistochemistry

Immunohistochemical analysis was carried out by using the primary antibodies reported in Table 1. In detail, mouse monoclonal anti-tyrosine hydroxylase (TH) primary antibody (Sigma) was used at 1:1000, mouse monoclonal anti-glial fibrillary acidic protein (GFAP) primary antibody (Sigma) was used at 1:400, mouse monoclonal anti-Bax primary antibody (Santa Cruz Biotechnologies, CA, USA) was used at 1:100. Sections were de-waxed by immersion in xylene and re-hydrated by using decreasing alcohol solutions. After permeabilization with 0.1% Triton X in PBS for 10 min and inhibition of endogenous peroxidases by 3% hydrogen peroxide in PBS for 30 min, sections were incubated with 10% normal goat serum in PBS for 1 h at room temperature and then transferred into solutions containing primary antibodies and 2% normal goat serum in PBS overnight at 4°C. The antigen-antibody reaction was revealed using biotin-conjugated secondary antibodies (1:200, Vector Laboratories, Burlingame, CA, USA; Table 1) for 2 h, followed by exposure to avidin-biotin complex (ABC, Vector) for 1 h and the peroxidase substrate diaminobenzidine (DAB, Vector). The time in DAB substrate was fixed at 3 min for all immunohistochemical reactions.

**Table 1:** Primary and secondary antibodies used for immune-histochemistry.

Primary antibodies	Host	Clone identity	Product code	RRID	Purchaser
Monoclonal anti-tyrosin hydroxylase	Mouse	TH-2	T1299	AB_477560	Sigma-Aldrich
Monoclonal anti-glial fibrillary acidic protein	Mouse	G-A-5	G3893	AB_477010	Sigma-Aldrich
Monoclonal anti-Bax	Mouse	B-9	SC-7480	AB_626729	Santa Cruz Biotechnology
**Secondary antibodies**	**Host**	**Lot number**	**Product code**	**RRID**	**Purchaser**
Biotynilated anti-mouse IgG (H + L)	Goat	T1031	BA-9200	AB_2336171	Vector Laboratories

Sections from Bax and nigral TH immunohistochemistry were counterstained by plunging in Haematoxylin solution (Sigma) for 2 min.

All sections were dehydrated in increasing alcohol solutions, clarified with xylene and coverslipped with DPX (Sigma).

Sections from each experimental group were equally represented during the same set of immunohistochemical analysis.

For each rat, Bax immunopositive cells from Cornu Ammonis (CA) were counted in three consecutive hippocampal sections spaced about 80 μm and obtained starting at approximately 2.5 mm posterior to bregma (Paxinos and Watson, 1986). Similarly, four 80 μm-spaced consecutive sections were stained for GFAP immunohistochemistry. Cell counts were also carried out in substantia nigra, where the number of TH-positive cells was evaluated within three consecutive sections, spaced about 80 μm, and collected starting at about 5.5 mm posterior to bregma (Paxinos and Watson, 1986). Finally, for each rat 10 consecutive striatal sections, spaced about 80 μm, were collected starting approximately at 1.4 mm anterior to bregma (Paxinos and Watson, 1986) and alternatively assigned to TH and GFAP immunostaining and related densitometric analysis.

All sections were analyzed under Nikon Eclipse 80i light microscope, equipped for image analysis software (Nikon, Tokyo, Japan).

Densitometric analysis was carried out by using NIH IMAGEJ 1.61. Optical density (OD) for TH immunoreactivity in the striatum was measured at a magnification of ×2 at a resolution of 300 dpi. TH-Positive striatum profile was drawn according with the rat atlas (Paxinos and Watson, 1986). In detail, for each section the OD was obtained as the difference between the average pixel density of the TH-positive striatum (automatically provided by the software) and the surrounding TH-negative area (corresponding to the thin subcortical white matter). This allowed to avoid bias due to differences in the background among different images. For each animal the final OD value was obtained as the mean ± SEM of 10 values. Densitometric analysis of GFAP within striatum and hippocampus represents the difference between the average density per pixel of GFAP-positive areas and GFAP-negative areas (background). In detail, due to non-homogeneous staining for GFAP, as GFAP-positive area we considered a rectangle of 450 μm × 350 μm selected within each GFAP-positive striatal or hippocampal sub-region (namely, CA3 and CA4), whereas as GFAP-negative area we considered three round-shaped small areas, which were not stained and which were placed in specific spots close to positive areas within the hippocampal region. For each image, the average of the three OD values related to such GFAP-negative areas was considered as the background.

### Statistical Analysis

For Comet Assay Multifactor Analysis of Variance (MANOVA) was used to assess the significance of factor effects such as: animals, slides and noise exposures. For statistical analysis the software SGWIN (Windows 98) was used. As indicated by a panel of experts the animal is the recognized unit for *in vivo* studies with Comet assay (Hartmann et al., 2003; Lovell and Omori, 2008) and at least four animal of a single gender are suggested to be included in each dose group at each sample time (Tice et al., 2000). In the present study the statistical unit is the animal. Four animals per group were used and group summaries ±SD are presented.

For catecholamine assay a standard curve was prepared using known amounts of DA, noradrenaline (NA) and metabolites (Sigma) dissolved in 0.1 M PCA containing a constant amount (10 pg/μl) of the internal standard diaminobenzylamine (Fornai et al., 1997). The standard curve for each compound (DA, NA and 3,4-dihydroxyphenylacetic acid, DOPAC) was calculated using regression analysis obtained by plotting ratios of the peak areas (compound area/ diaminobenzylamine area) for various concentrations of each compound recorded at the reducing electrode. An analogous regression analysis was performed for the oxidizing electrode to provide a further validation of the consistency of the measurements. Since the signal was cleaner (less artifacts) at the reducing electrode, we just used this latter one to calculate the final data.

For NA, DA and DOPAC levels, results are expressed as the mean ± SEM of 10 values per experimental group. The effects of noise on catecholamine levels were statistically evaluated using analysis of variance with Sheffè’s *post hoc* analysis. The null hypothesis was rejected when *p* ≤ 0.05.

Cell counts within substantia nigra slices were reported as the mean number ± SEM of TH positive cells counted in each group. Hippocampal Bax-positive cells were reported as the mean value ± SEM of stained cells counted in each group. Finally, the OD of TH-positive striatal sections and GFAP-positive striatal and hippocampal sections was expressed as the mean percentage ± SEM.

*F*-values and degrees of freedom (df) related to all the experimental measures were provided (Table 2).

**Table 2:** *F*-values and degrees of freedom related to all experimental data.

		*F*-values	Degrees of freedom
Comet assay	Cerebellum	67.02	1
	Hippocampus	41.44	1
	Striatum	22.42	1
Diffusion assay	Cerebellum	2.58	1
	Hippocampus	0.84	1
	Striatum	1.04	1
Biochemical assays	Striatal DA	101.470	9
	Striatal DOPAC	9.982	9
	Hippocampal DA	1.847	9
	Hippocampal NA	15.920	9
	Cerebellar NA	25.763	9
Cell counts	Nigral TH-positive cells	2.196	11
	CA Bax-positive cells	102.826	11
	CA1 Bax-positive cells	4.376	11
	CA2 Bax-positive cells	3.349	11
	CA3 Bax-positive cells	81.843	11
	CA4 Bax-positive cells	216.619	11
Optical density	Striatal TH	48.981	39
	Striatal GFAP	45.740	39
	CA1 GFAP	2.857	15
	CA2 GFAP	3.074	15
	CA3 GFAP	35.389	15
	CA4 GFAP	29.168	15

*CA, Cornu Ammonis; DA, dopamine; DOPAC, 3,4-dihydroxyphenylacetic acid; GFAP, glial fibrillary acidic protein; NA, noradrenaline; TH, Tyrosine hydroxylase*.

Inferential statistics for immunohistochemistry was carried out using ANOVA followed by *post hoc* Games-Howell test to compare control rats and rats sacrificed at 24 h and 7 days after loud noise exposure. The null hypothesis was rejected when *p* ≤ 0.05.

## Results

### DNA Integrity

A statistically significant increase of DNA damage was observed in all three brain areas immediately after the cessation of loud noise. In particular, all three parameters being selected increased significantly (*p* < 0.001). This refers to: (i) the % of DNA migrated into the tail; (ii) TL; and (iii) TM, as shown in Figure 3. Looking at the % of DNA migrated into the tail, which is considered the most objective one (Kumaravel and Jha, 2006), a statistically significant increase of DNA damage was observed both in cerebellum (*p* < 0.001; *F*-value = 67.02; df = 1), hippocampus (*p* < 0.001; *F*-value = 41.44, df = 1) and striatum (*p* < 0.001; *F*-value = 22.42, df = 1) immediately after loud noise exposure as shown in Figure 3. Cell count did not reveal the occurrence of any cell death. These findings allow to rule out that the amount of strand breaks observed in the present study might be due to a loss of DNA integrity related to cell death, thus indicating an authentic genotoxic effect. Strand breaks produced by noise exposure were comparable in all brain areas suggesting a rather general effect independently by neuronal phenotypes.

### Diffusion Assay

The results of the diffusion assay did not reveal the occurrence of apoptotic cells either in exposed or control animals, soon after the cessation of the stimulus, thus confirming the absence of apoptotic cell death found at light microscopy (cerebellum *p* = 0.17, *F*-value = 2.58; hippocampus *p* = 0.39, *F*-value = 0.84; striatum *p* = 0.35, *F*-value = 1.04, Table 2). These findings allow to rule out that strand breaks observed in the present study might be partly due to loss of DNA integrity related to cell death, supporting the authentic genotoxic effects of loud noise. Incidentally, these data led to reconsider the significance of Bax immunostaining reported later at light microscopy.

### Catecholamines Levels

**Figure 4:**
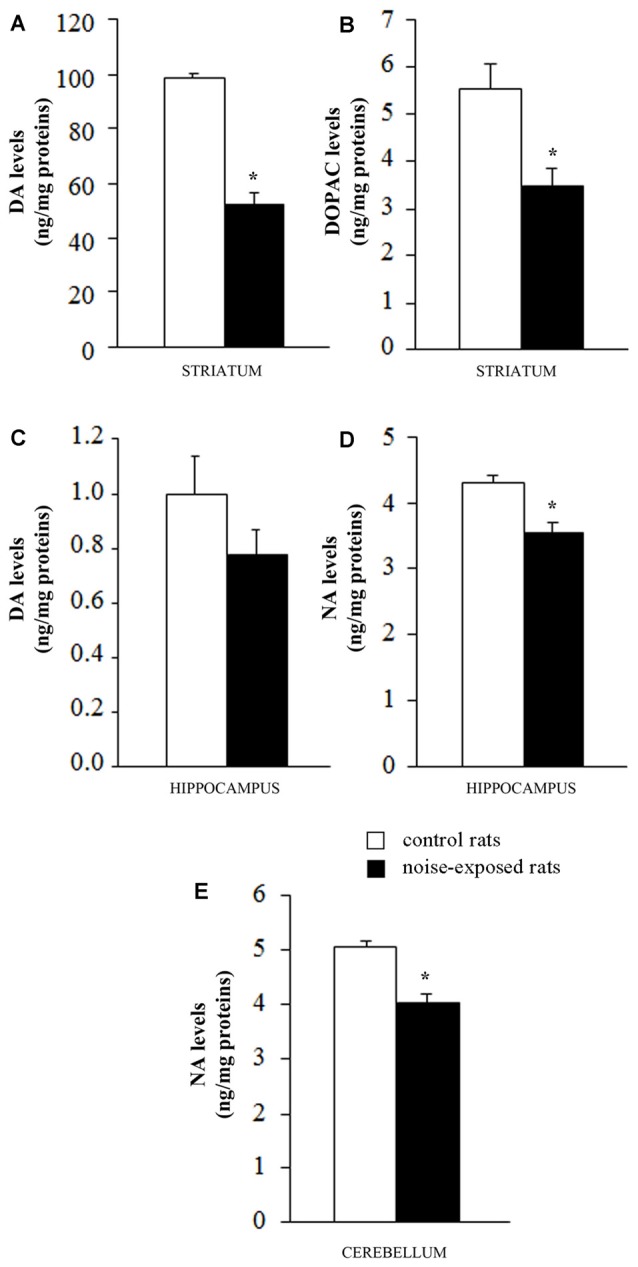
Loud noise exposure produces long-lasting (7 days), site-specific catecholamine loss. Graphs show catecholamine levels measured after noise exposure in specific brain areas: **(A)** dopamine (DA) and **(B)** 3,4-dihydroxyphenylacetic acid (DOPAC) levels in striatum; **(C)** DA levels in hippocampus; **(D)** noradrenaline (NA) levels in hippocampus and **(E)** cerebellum. White column = control rats; black column = 12 h noise-exposed rats sacrificed 7 days after exposure. *p ≤ 0.05 compared with controls.

When DA was measured in the striatum of control rats the quantitative level was similar to that reported in the literature for this rat strain (98.9 ± 1.4 ng/mg of protein, see for instance Fornai et al., 1996). This amount of DA was the highest catecholamine value between the various brain areas and it exceeded by far that measured in the hippocampus of control rats (1.0 ± 0.1; Figure 4A). Remarkably, loud noise produced a persistent reduction in striatal DA levels (66.4 ± 3.9) measured at 7 days after exposure (*F*-value = 101.470, df = 9; Figure 4A). At striatal level the reduction of DA was accompanied by a significant decrease of its intracellular metabolite DOPAC (3.5 ± 0.4 compared with 5.5 ± 0.5 of controls, *F*-value = 9.982, df = 9; Figure 4B). Unexpectedly, the loss of DA and DOPAC was comparable. In general, when a damage to nigro-striatal terminals occurs DOPAC levels are less reduced than DA concentration. This is expressed by an increase in the turnover index (DOPAC/DA). Such an index is generally reported to reflect a compensatory mechanism due to an increase in the DA synthesis which occurs in a partially DA-denervated striatum (Gesi et al., 2001). However, in the present experimental condition the loss of DA and DOPAC were comparable. In the hippocampus we measured a slight DA decrease (0.8 ± 0.1) which did not reach statistical significance (*F*-value = 1.847, df = 9; Figure 4C). In the hippocampus DOPAC was not detected either in control or in noise-exposed rats. When NA content was measured, a significant loss, was recorded both in the hippocampus (*F*-value = 15.920, df = 9) and cerebellum (*F*-value = 25.763, df = 9) of noise exposed-rats compared with controls (Figures 4D,E, respectively). It is noteworthy that baseline NA levels measured in the hippocampus were higher compared with DA levels (roughly 5-fold) and they were similar to cerebellar NA as reported in the literature for this rat strain (Fornai et al., 1996).

### Immunohistochemistry

**Figure 5:**
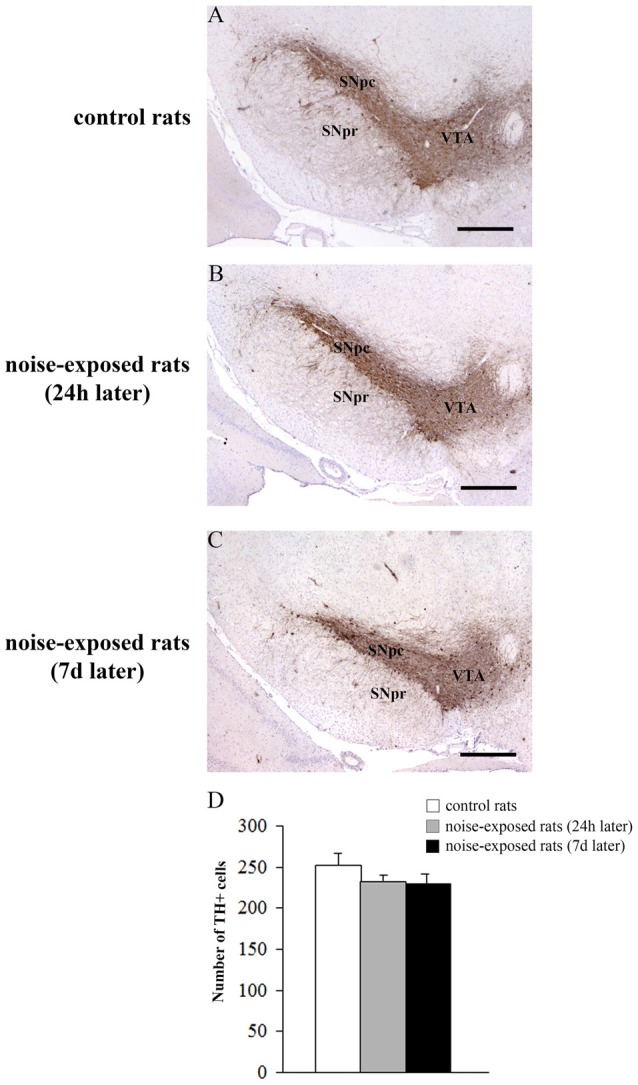
Loud noise does not reduce tyrosine hydroxylase (TH)-positive cell bodies in the substantia nigra. Representative pictures of TH immunopositive sections of substantia nigra of a control rat **(A)** and rats sacrificed at 24 h **(B)** or 7 days **(C)** after 12 h noise exposure. The number of TH-positive cells counted in three non-consecutive sections (see “Materials and Methods” Section) is reported in the graph **(D)**. White column = control rats; gray column = 12 h noise-exposed rats sacrificed 24 h after exposure; black column = 12 h noise-exposed rats sacrificed 7 days after exposure. SNpc, substantia nigra pars compacta; SNpr, substantia nigra pars reticulata; VTA, ventral tegmental area. Bar = 500 μm.

**Figure 6:**
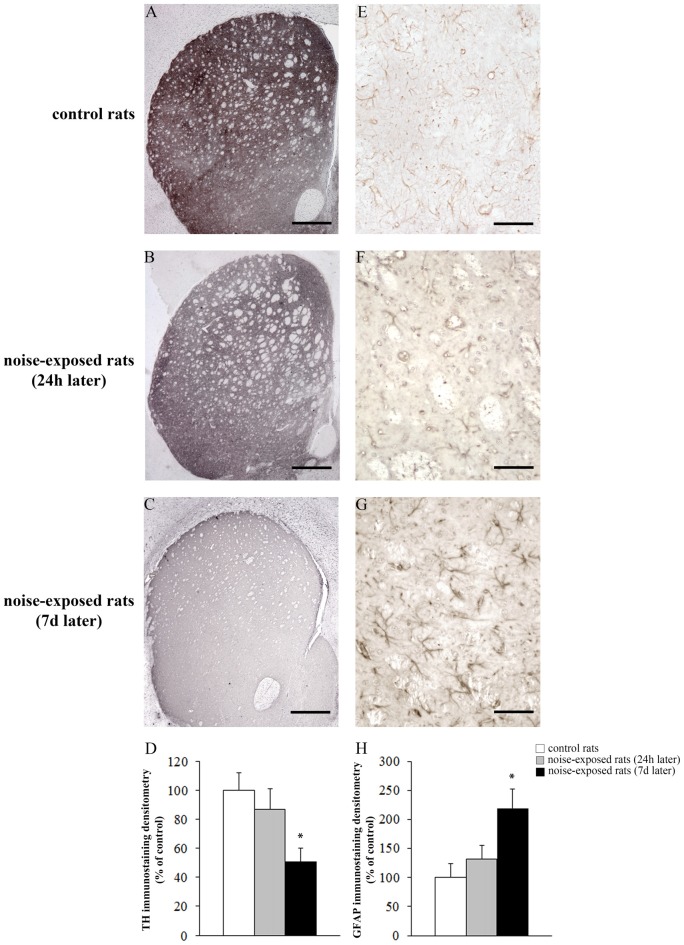
Loud noise exposure reduces striatal catecholamine axons and increases glial fibrillary acidic protein (GFAP). Representative striatal TH immunostaining from: control rat **(A)** and rats sacrificed 24 h **(B)**, and 7 days **(C)** after 12 h of loud noise exposure. Densitometric analysis of TH-immunostaining is reported in the graph **(D)**. Representative striatal GFAP immunostaining from: control rat **(E)** and rats sacrificed 24 h **(F)** and 7 days **(G)** after 12 h of loud noise exposure. Optical density (OD) of GFAP-immunostaining is reported in the graph **(H)**. White column = control rats; gray column = 12 h noise-exposed rats sacrificed 24 h after exposure; black column = 12 h noise-exposed rats sacrificed 7 days after exposure. *p ≤ 0.05 compared with controls. Bars = **(A–C)** 660 μm; **(E–G)** 70 μm.

Qualitative analysis of TH-positive neurons did not show any significant alteration in substantia nigra pars compacta (SNpc) of noise exposed rats both at 24 h and 7 days after noise exposure (Figures 5A–C). This was confirmed by cell counts carried out in TH-stained non-consecutive sections, which revealed the absence of any significant cell loss in the substantia nigra of noise-exposed rats (Figure 5D, *F*-value = 2.196, df = 11). Conversely, striatal TH immunostaining is slightly reduced at 24 h, while it was markedly reduced at 7 days after noise exposure (Figures 6A–C). This was also confirmed by comparing the OD related to TH immunostaining measured in the striatal sections of control and noise-exposed rats (Figure 6G, *F*-value = 48.981, df = 39). The loss of striatal TH immunostaining was accompanied by an increase in GFAP immunostaining (Figures 6D–F), which became significant at 7 days after noise exposure, as indicated by the OD measures (Figure 6H, *F*-value = 45.740, df = 39).

**Figure 7:**
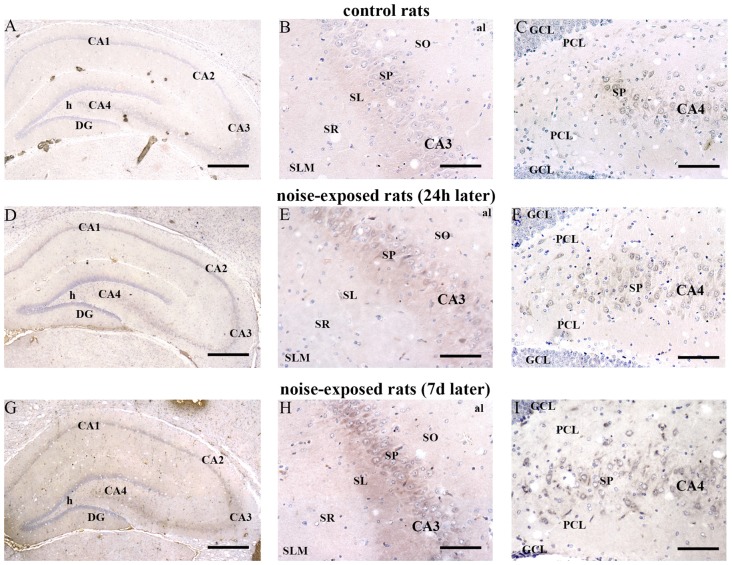
Loud noise increases Bax in the hippocampus. Representative pictures of hippocampal Bax immunostaining from control **(A–C)** and noise-exposed **(D–I)** rats. Pictures at low magnification show hippocampal Bax immunostaining of a control rat **(A)** and rats sacrificed at 24 h **(D)** and 7 days **(G)** after loud noise exposure. Images at a higher magnification show CA3 and CA4 sub-regions from a control rat (**B,C**, respectively) and rats sacrificed at 24 h (**E,F**, respectively) and 7 days (**H,I**, respectively) after loud noise exposure. al, alveus; CA, Cornu Ammonis; DG, dentate gyrus; GCL, granule cell layer; h, hylus; PCL, polymorphic cell layer; SL, stratum lucidum; SLM, stratum lacunosum-moleculare; SO, stratum oriens; SP, stratum pyramidale; SR, stratum radiatum. Bars = **(A,D,G)** 500 μm; **(B,C,E,F,H,I)** 89 μm.

**Figure 8:**
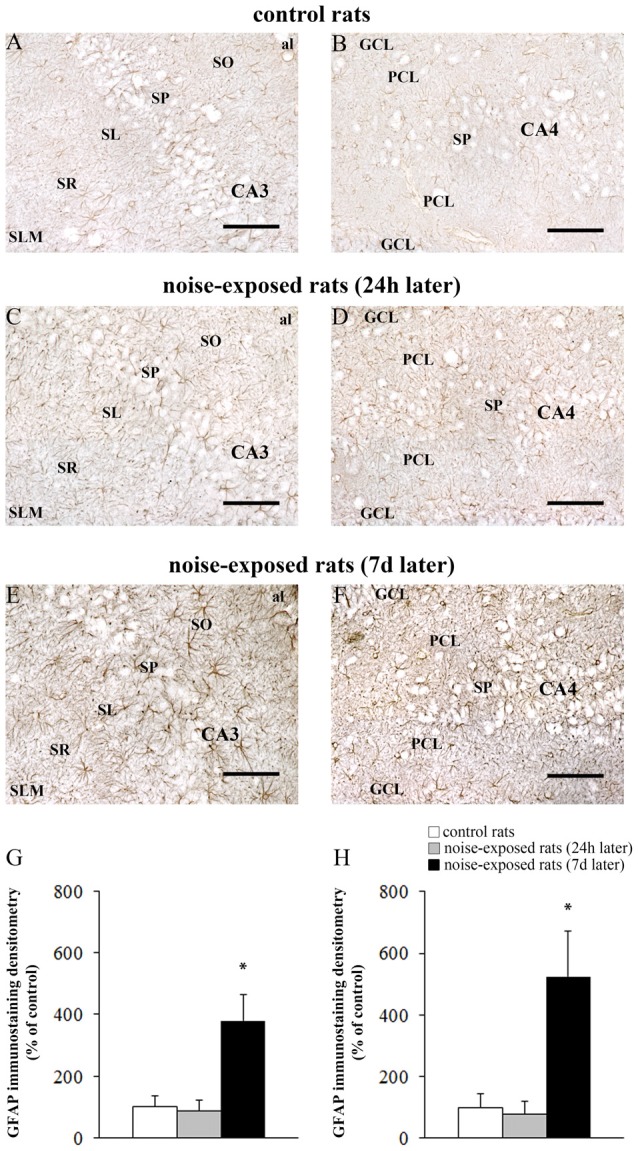
Loud noise increases GFAP in the hippocampus. Representative pictures of hippocampal GFAP immunostaining in the **(A)** CA3 and **(B)** CA4 sub-regions of a control rat, **(C)** CA3 and **(D)** CA4 of a rat sacrificed 24 h after noise exposure and **(E)** CA3 and **(F)** CA4 of a rat sacrificed 7 days after noise exposure. The graphs **(G,H)** report the OD measures related to GFAP-immunostaining in CA3 and CA4, respectively. al, alveus; CA, Cornu Ammonis; DG, dentate gyrus; GCL, granule cell layer; h, hylus; PCL, polymorphic cell layer; SL, stratum lucidum; SLM, stratum lacunosum-moleculare; SO, stratum oriens; SP, stratum pyramidale; SR, stratum radiatum. *p ≤ 0.05 compared with controls and noise-exposed rats (24 h). Bars = 89 μm.

In hippocampus, Bax immunopositivity appeared slightly increased in noise-exposed rats compared with controls (Figures 7A–I). Such an increase involved scattered hippocampal regions, mainly at the level of pyramidal cells of the CA in the CA3 and CA4 areas (Figures 7B,C,E,F,H,I, respectively). Since the high cellular density of pyramidal cells lying in the CA, the difficulty to distinguish the cell contour might bias the evaluation of positive cells. In fact, we considered in the count only those cells with clearly visible nucleus and whole contour, despite this led to an underestimation of Bax positive neurons in the hippocampus (*F*-value = 102.826, df = 11). Nonetheless, Bax-positive cells in rats sacrificed 7 days after noise exposure significantly increased compared with controls (436 ± 11.2 and 167.3 ± 23.7, respectively). No significant effect was produced in rats sacrificed after 24 h (224.6 ± 32.7). A similar trend was found when the number of Bax-positive cells counted within each hippocampal CA sub-region was compared, as reported in Table 3. Finally, a scattered increase in GFAP immunostaining, which appears to be mainly localized in the CA3 (Figures 8A,C,E) and CA4 regions (Figures 8B,D,F) was found in the hippocampus of rats sacrificed at 7 days after noise exposure.

**Table 3:** Number of Bax-positive cells in hippocampal CA.

	CA	CA1	CA2	CA3	CA4
Control	167.3 ± 23.7	27.6 ± 4.1	23.2 ± 4.5	90.8 ± 12.9	25.8 ± 3.2
Noise-exposed rats (24 h)	224.6 ± 32.7	35.3 ± 3.8	23.9 ± 3.3	126.4 ± 19.4	39.1 ± 7.9
Noise-exposed rats (7 days)	436.8 ± 11.2*	40.6 ± 2.3	31.6 ± 3.3	236.8 ± 6.7*	127.8 ± 5.9*

*p ≤ 0.05 compared with controls and noise-exposed rats (24 h).

**Figure 9:**
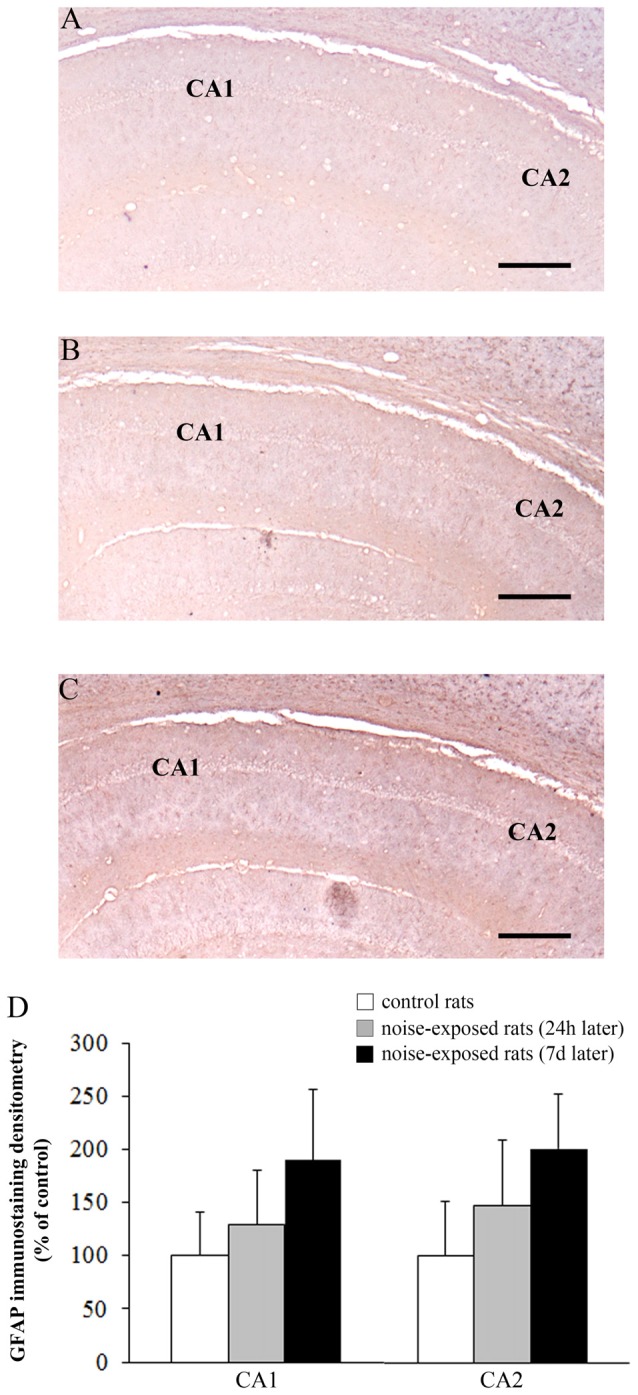
Loud noise does not change GFAP immunostaining in hippocampal CA1 and CA2. Representative pictures of GFAP immunostaining within hippocampal CA1-CA2 subregions of a control rat **(A)** and rats sacrificed at 24 h **(B)** or 7 days **(C)** after 12 h noise exposure. OD related to GFAP immunostaining within CA1 and CA2 **(D)** shows that noise exposure does not modify GFAP immunoreactivity within these hippocampal subregions. Bar = 300 μm.

The OD values related to GFAP immunostaining in CA3 and CA4 sub-regions are reported in the graphs of Figure 8G (*F*-value = 35.389, df = 15), and Figure 8H (*F*-value = 29.168, df = 15), respectively. In Figure 9 the OD of GFAP in CA1 (*F*-value = 2.857; df = 15) and CA2 (*F*-value = 3.074; df = 15) hippocampal sub-regions were also reported.

## Discussion

This study demonstrates that loud noise exposure produces a damage to different brain areas, as assessed by genetic, biochemical and morphological analysis.

In particular, a significant loss of DNA integrity in three critical brain areas (striatum, hippocampus, cerebellum) of rats exposed for 12 h to 100 dBA loud noise was observed. The occurrence of an authentic damage is validated by multiple experimental approaches. This occurs frankly at the level of striatal nerve terminals, while it is not consistently evident in the cell bodies. In fact, even in keeping with the Comet assay, we can rule out that the elevation of DNA strand breaks was due to cell death, and the diffusion assay allowed to rule out a cell death-related DNA damage; moreover, light microscopy failed to document substantial cell loss at 24 h after noise exposure. The occurrence of Bax positive cells in the hippocampus is in sharp contrast to the results produced by the diffusion assay. This may be due to: (i) the count of non-neuronal cells in the diffusion assay which are way more abundant compared with neurons thus diluting the occurrence of a slight apoptosis which is suggested by immunohistochemical analysis; (ii) the lack of a real apoptosis in the presence of Bax immunopositivity which may occur under specific experimental conditions; (iii) it is very likely that both phenomena concur to generate these discrepancies; and (iv) the different timing between Bax immunostaining and diffusion assay. On the other hand, when we performed Bax immunostaining at 24 h after noise exposure we failed to find any differences in comparison with controls. This is in line with diffusion assay data, obtained soon after the cessation of the noise stimulus. An increase in the pro-apoptotic proteins Bax and caspase-3 as well as the anti-apoptotic protein Bcl-2 was previously reported in the hippocampus of rats exposed to loud noise (Kim et al., 2013). These findings indicate that noise is able to modulate the apoptotic pathway, producing a final effect, which depends on the intensity and/or timing of exposure.

Remarkably, at 7 days after noise exposure we measured a loss of striatal DA terminals, which was substantiated both by a decrease in DA and DOPAC levels and a loss of TH immune-staining and increase in GFAP immunostaining. In contrast, we failed to document a loss of TH immunopositive DA cell bodies in the SNpc, which is the main source of striatal DA. At this time, after loud noise exposure, we show significant hippocampal alterations. These consist of a significant loss of NA innervation as witnessed by quantitative measurement of hippocampal NA levels, a slight decrease in hippocampal DA levels and an increase in GFAP immunostaining. If measuring DA and NA levels represents a solid index of integrity of hippocampal catecholamine innervation, the significance of GFAP immune-staining is more elusive. GFAP immunostaining is routinely carried out as a indirect index of neurotoxicity, although other mechanisms may produce GFAP variations. On the other hand, the OD measure that we carried out in GFAP-stained slices does not provide absolute quantitative measurement, due to a lack of a steady linear correlation between the OD values and the level of expression of a specific antigen. Moreover in the hippocampus of noise-exposed rats we detected an increase in Bax positive cells within Cornu Ammmonis (CA3 and CA4 subfield) as discussed above. Finally, in the cerebellum, a significant decrease in NA levels was measured at 7 days after noise exposure.

The same intensity and duration of noise exposure (100 dBA for 12 h) which was applied in the present study, has been previously shown to be effective in inducing genotoxic effects in peripheral extra-auditory organs such as the myocardium and adrenal gland of the rat (Lenzi et al., 2003; Frenzilli et al., 2004).

Results on DNA damage provided in the present study might be due either to a clastogenic effects of oxyradicals and/or DNA repair of oxidized bases, which implies the expression of alkali labile sites, detected by the alkaline Comet assay. In line with this, analogous findings that we observed on DNA following loud noise in peripheral organs were associated with an increase in reactive oxygen species (ROS; Lenzi et al., 2003). Similarly, in the cochlea, which is directly involved in noise transmission, ROS levels were found the be significantly elevated already at 1 h after exposure to 110 dB noise and persisting after noise exposure (Ohlemiller et al., 1999).

Concerning the effects of noise on ROS within brain areas, an increase in cerebellar ROS levels and in hippocampal catalase activity were described in rats exposed for 2 h to 95–97 dB (Uran et al., 2010). Similarly, increased lipid peroxidation and increased activity of superoxide dismutase, glutathione peroxydase, catalase and acetylcholinesterase, were found in association with a decrease in glutathione levels in the hippocampus from rats exposed to 100 dBA for 4 days (Manikandan et al., 2006). Chronic impairment of spatial and associative memory was observed by Uran et al. (2010) in noise-exposed rats. These authors suggested that such an effect was dependent on the imbalance of oxidative status in hippocampus and cerebellum, which were involved in memory processing.

The strong association between ROS elevation and DNA toxicity is well-known (Cross et al., 1987; Lemasters et al., 1992). In particular, ROS produce an oxidative damage of DNA which undergoes single-strand breaks and inter/intra-strand crosslinks (Caraceni et al., 1997). For instance, malondialdehyde (MDA) produced lipid peroxidation was supposed to be responsible for cellular toxicity by cross-linking protein and nucleic acids after noise exposure in the hippocampus (Cheng et al., 2011). Van Campen et al. (2002) reported an elevation of 8-hydroxy-2′-deoxyguanosine (8OHdG) in brain and liver (besides the dramatic effects on the cochlea) of rats exposed to loud noise (120 dB). Thus, ROS are likely to play a causal role in the genetic damage produced by loud noise exposure both in the brain and peripheral organs. According to these findings, the association between noise exposure, oxidative processes and persistent DNA damage deserves further attention due to the potential detriment of a long-lasting DNA damage up to mutagenesis (Preston-Martin et al., 1989; Emerit, 1994; Hours et al., 2009).

Previous studies have shown that noise exposure induces behavioral and autonomic alterations which represents the so called “stress response” (Van de Kar and Blair, 1999). An important component of such a stress response is represented by increased NA release peripherally and within the brain (Tanaka et al., 2000). Similarly, stress-induced brain DA release has been reported, and release of brain catecholamines in response to noise stress is well documented (Ravindran et al., 2005). In line with this Samson et al. (2007) showed an increase of NA in whole brain samples of rats after different times of 100 dB noise exposure. In contrast, a decrease in brain NA levels was described soon after acute noise exposure (Okada et al., 1983). This discrepancy may depend on the depleting effects, which occur immediately after NA release. This effects is expected to persist when noise exposure occurs intermittently for prolonged time intervals. This may explain why a significant decrease of NA was reported in the hippocampus of rats chronically exposed to both 80 and 100 dB, which lasted until 30 and 40 days after the cessation of the noise stimulus (Chengzhi et al., 2011). The persistency of NA depletion followed by a complete recovery would correspond to the time window required to compensate for a reiterated NA release which exhausted NA stores.

Despite a strong association between noise exposure, ROS and DNA damage is demonstrated, when considering system neuroscience the fundamental question which needs to be answered concerns deciphering which anatomical pathway connects noise exposure and the inner ear with various brain regions. The only gateway for noise to influence internal organs of the cell body including specific brain areas is provided by the transduction of sound into action potentials at the level of the inner ear. This phenomenon is essential to allow the transmission of loud noise from the external environment to the brain and from the brain to peripheral organs. Thus, the specific synaptic network of auditory pathways is essential to translate the effects of loud noise exposure to the human body. When examining the complex synaptology of the inner ear it is known that acoustic stimulation, despite being canonically transmitted to the thalamus to reach the primary auditory cortex, it also impinges on the conundrum of several nuclei of the brainstem which constitute the reticular formation (Yeomans and Frankland, 1995; Carlsen, 2015). In fact, in a recent manuscript Carlsen (2015) demonstrated that a white (broadband) noise is much more effective in producing a startle response compared with a pure tone owing the same intensity. The startle response is based on the activation from the inner ear of cochlear nuclei projecting to non-canonical auditory pathways merging in the reticular formation. In detail, loud noise compared with loud tones are much more effective in activating the gigantocellular reticular nucleus (Yeomans and Frankland, 1995). This is likely to depend on the fact that white (broadband) noise acts on a greater surface of the basilar membrane (Hudspeth, 2000), which in turn determines the excitation of a higher number of auditory cells, which spread their excitation on a greater number of reticular neurons where these excitatory signals converge. One major output from the dorsal cochlear nucleus to the reticular formations consists in excitatory fibers to the pontine nucleus of Locus Coeruleus (LC; Kaltenbach, 2006). Thus, it is likely that a powerful excitotoxic effect on LC neurons may be detrimental for NA axon survival. This may explain reduced NA levels we found both in cerebellum and hippocampus which are compatible with the NA projections from LC (Fornai et al., 1996). Similarly, the DA neurons of the SNpc and ventral tegmental area (VTA) receive a powerful excitatory input indirectly from auditory pathways which may explain the powerful detrimental effects of loud noise on these DA containing nuclei projecting to the striatum and hippocampus (Gale et al., 2008).

Thus, although a reduction of NA and DA levels we observed chronically in rats exposed to noise was somehow unexpected at first, an in depth analysis of noise transmission pathways poses it into a logical chain of events. The activation of catecholamine-containing reticular nuclei is key both for brain catecholamine pathways and for vegetative effects induced by loud noise exposure. In fact, if increased activity of LC reticular neurons projecting to the forebrain is supposed to promote arousal and attention (Kaltenbach, 2006), the descending fibers from the brainstem reticular formation explain why noise exposure induces those autonomic alterations contributing to the myocardial toxicity induced by loud noise (Tanaka et al., 2000; Lenzi et al., 2003) and to deleterious effects of noise on the adrenal gland which is mediated by the vegetative nervous system and its connections with the hypothalamo-pituitary axis (Frenzilli et al., 2004). It appears that the catecholamine core of the brainstem reticular formation mediates both effects of loud noise on peripheral organs and the behavioral alterations produced by specific brain regions, which receive the ascending reticular catecholamine pathways arising from both NA- and DA-containing nuclei. Thus, it may be hypothesized that an excess of excitation of these catecholamine-containing nuclei may lead to long-lasting deleterious effects. In fact it is well-known that the amount of glutamate which is released by the acoustic projections may produce excitotoxicity (Chen et al., 2009). The overactive excitotoxic auditory input to brainstem catecholamine reticular nuclei may produce the loss of striatal DA as well as hippocampal and cerebellar NA axons. In general, alterations in brain monoamines in response to noise is well documented (Ravindran et al., 2005). The reduced DA levels that we found within the striatum of rats exposed to 12 h of noise confirm that loud noise increases the vulnerability of nigrostriatal projections. This is in line with previous studies showing that, a short noise exposure (for 6 h), although leaving intact striatal DA levels *per se*, drastically worsen striatal DA depletion and the loss of striatal DA axons induced by MDMA (Gesi et al., 2004). Our present data confirm what published by Hu et al. (2014), who found a decrease of striatal DA in rats exposed to loud noise. Interestingly, as mentioned in the Results section, we failed to document a compensatory increase in the turn-over index (DOPAC/DA) which normally occurs as a compensatory effect (Yurek et al., 1989; Sedelis et al., 2000; Gesi et al., 2001). This finding remains unexplained but it could be related to a decreased activity of spared DA neurons. This hypothesis would also explain what found by Tsai et al. (2005) who measured a concomitant decrease of DA and DOPAC in striatal dialysate of rats exposed to noise stress. In the present study, we documented also a decrease in hippocampal and cerebellar NA. In line with this, a decrease in brain NA was described after noise (Okada et al., 1983), and a significant decrease of NA was reported in the hippocampus of rats chronically exposed to both 80 and 100 dB, which was long-lasting since it persisted up to 30 and 40 days after the cessation of the noise stimulus (Chengzhi et al., 2011). The intriguing hypothesis that those pathways recruited by loud noise exposure produce a decrease of TH activity may explain both trivial effects on monoamine levels which occurs along with the unexpected decrease in turnover ratio (DOPAC/DA).

DA neurons projecting to the cochlea were described within the lateral division of the superior olivary complex in the guinea pig (Gáborján et al., 1999; Mulders and Robertson, 2004). DA neurons mainly innervate the inner hair cells and they exert a tonic inhibition of auditory nerve activity, thus preserving auditory sensitivity and protecting cochlear hair cells against excitotoxicity (Mulders and Robertson, 2004; Niu et al., 2007; Maison et al., 2012).

Interestingly, a loss of hearing was recently described as a non-motor symptom in Parkinson’s disease (Vitale et al., 2012; Lai et al., 2014). In detail, auditory impairment in parkinsonian patients, measured through oto-acoustic emission recording and pure-tone audiometry, are improved by L-DOPA treatment. L-DOPA-induced auditory improvement is consistent with improvement of motor symptoms along an overlapping dose-response curve (Pisani et al., 2015).

The potential damage of cochlear efferent DA fibers might contribute *per se* to hearing loss observed in Parkinson’s disease. On the other hand, it was demonstrated that mesencephalic DA neurons, mainly located within VTA, receive a strong innervation from dorsal and ventral cochlear nuclei (Herbert et al., 1997). Then, from VTA the auditory stimuli are widely projected to several CNS areas via mesocortical, mesolimbic and mesostriatal pathways. These projections are even more widespread if one takes into account the isodendritic morphology of the DA neurons and their bipolar axons, as typical reticular neurons.

Concerning NA cochlear innervation, a reduction in NA concentrations was described in the cochlea of rats acutely exposed to noise (Vicente-Torres and Gil-Loyzaga, 1999). It is likely that these NA fibers derive from NA brainstem nuclei, such as LC, and/or from the superior olivary nucleus. In the rat olivo-cochlear neurons receive NA innervation, arising from LC, thus demonstrating the occurrence of functional connections between LC and the auditory system (Kromer and Moore, 1980; Mulders and Robertson, 2001, 2005). On the other hand, LC is known to be prone to different kinds of stressful stimuli (George et al., 2013). Therefore, the loss of NA content that we found in the hippocampus and cerebellum after noise exposure might depend on noise-induced impairment of the LC efferent projections. Remarkably, decreased levels of NA within hippocampus which were measured following loud noise exposure, occur both in mood disorders and cognitive impairment (Fuchs et al., 2004; Ramos and Arnsten, 2007). In line with this, depressive syndromes have been recently associated with noise exposure (Orban et al., 2016).

In fact, experimental findings in animal models provided a potential mechanistic explanation showing that both acute and chronic noise exposure induce transient or persistent alterations, focally, within specific CNS areas (Ising and Braun, 2000; Manikandan et al., 2006; Goble et al., 2009). For instance, in rodents exposed to loud noise persistent cognitive and memory impairment were observed in association with tau phosphorylation and neuronal apoptosis both in dentate gyrus (DG) and CA of the hippocampus. This hippocampal pathology was related to altered monoamine and amino-acidic hippocampal innervation (Cui et al., 2013; Busceti et al., 2015; Cheng et al., 2016).

In noise-exposed rats, impairment in spatial and associative memory is accompanied by an imbalance between pro- and anti-oxidants in the hippocampus and cerebellum (Uran et al., 2010; Cheng et al., 2016; Sikandaner et al., 2017). Remarkably, the hippocampus, despite not belonging to the auditory pathways, was recently shown to be more vulnerable than the auditory cortex to environmental noise (Cheng et al., 2016). In particular, Cheng et al. (2011, 2016) hypothesized that noise-induced oxidative stress can be transmitted to hippocampus via ascendant lemniscal and inferior colliculus, thus causing memory impairment and pathological tau hyperphosphorylation. Manikandan et al. (2006) also reported that cognitive impairment related to noise-induced stress is associated with altered hippocampal neuronal dendritic function and abnormal tau phosphorylation, which in turn, might depend on the loss of cytoskeleton integrity.

In line with this we demonstrate the chronic hippocampal impairment produced by noise, we found an increase of Bax, a protein which is known to be involved in p53-mediated apoptotic pathway, and GFAP, which represents the marker of activated astroglia.

In conclusion, our study indicates that loud noise exposure represents a detrimental stimulus for specific brain areas. This consists mostly on decreased catecholamine innervation which involves multiple brain regions. Interestingly, these data lend substance to clinical findings showing impaired memory, mood alterations and other behavioral alterations induced by prolonged noise exposure. The occurrence of nigrostriatal DA innervation further strengthens this association. In fact, in Parkinson’s disease a loss of auditory function occurs, which is compatible with the loss of cochlear DA innervation, which in turn protects from the effects of loud noise. Thus, a vicious circle may occur, where the excitotoxic effects of loud noise may destroy DA nerve endings producing a loss of DA in their terminal fields, including the efferent synapses with cochlear hair cells, where DA exerts a gating control. In this way, the transmission of loud noise would no longer be hampered despite a loss in the detection of pure tones. Altogether our findings provide a bridge between environmental exposure to loud noise and the onset of neuropsychiatric alterations such as: cognitive impairment, depressive symptoms, behavioral abnormalities, movement disorders, as recently documented in general populations. Since environmental noise exposure represents an increasing worldwide polluting agent (WHO (World Health Organization), 2011), our data deserve particular attention in the light of their potential impact on public health.

## Author Contributions

FF and GF: study concept and design; GF, VS, EC, SC, MF, LR and PL:acquisition, analysis and interpretation of data; GF, FSG, MF and FF:drafting of the manuscript; GF, MF, AFalleni, AFrati, SG, FB, FSG and FF: critical revision of the manuscript; VS and GF: statistical analysis (supervised statistical analysis for comet and diffusion assay), EC, SC, MF (supervised statistical analysis forimmune-histochemistry and biochemistry); GF: obtained funding; FF: study supervision; VS, EC, AFalleni, SC, PL, FB, FSG and LR: technical assistance; AFalleni, GF, FSG, FF, MF, PL, VS, EC, SC, AFrati, SG, FB and LR: final approval of the version to be published.

## Conflict of Interest Statement

The authors declare that the research was conducted in the absence of any commercial or financial relationships that could be construed as a potential conflict of interest.

## References

[B1] Berglund B. Lindvall T. Schwela D. H. (1999). Guidelines for Community Noise. London: World Health Organization.

[B2] Busceti C. L. Di Pietro P. Riozzi B. Traficante A. Biagioni F. Nisticò R.. (2015). 5-HT_2C_ serotonin receptor blockade prevents tau protein hyperphosphorylation and corrects the defect in hippocampal synaptic plasticity caused by a combination of environmental stressors in mice. Pharmacol. Res. 99, 258–268. 10.1016/j.phrs.2015.06.017 26145279

[B3] Caraceni P. De Maria N. Ryu H. S. Colantoni A. Roberts L. Maidt M. L.. (1997). Proteins but not nucleic acids are molecular targets for the free radical attack during reoxygenation of rat hepatocytes. Free Radic. Biol. Med. 23, 339–344. 10.1016/s0891-5849(96)00571-0 9199897

[B4] Carlsen A. N. (2015). A broadband acoustic stimulus is more likely than a pure tone to elicit a startle reflex and prepared movements. Physiol. Rep. 3:e12509. 10.14814/phy2.12509 26311832 PMC4562592

[B5] Chen Z. Peppi M. Kujawa S. G. Sewell W. F. (2009). Regulated expression of surface AMPA receptors reduces excitotoxicity in auditory neurons. J. Neurophysiol. 102, 1152–1159. 10.1152/jn.00288.2009 19515954 PMC2724347

[B6] Cheng L. Wang S. H. Chen Q. C. Liao X. M. (2011). Moderate noise induced cognition impairment of mice and its underlying mechanisms. Physiol. Behav. 104, 981–988. 10.1016/j.physbeh.2011.06.018 21726571

[B7] Cheng L. Wang S. H. Huang Y. Liao X. M. (2016). The hippocampus may be more susceptible to environmental noise than the auditory cortex. Hear. Res. 333, 93–97. 10.1016/j.heares.2016.01.001 26773751

[B8] Chengzhi C. Yan T. Xuejun J. Xiang L. Youbin Q. Baijie T. (2011). Recovery of chronic noise exposure induced spatial learning and memory deficits in young male Sprague-Dawley rats. J. Occup. Health 53, 157–163. 10.1539/joh.l10125 21422718

[B9] Christensen J. S. Raaschou-Nielsen O. Tjønneland A. Overvad K. Nordsborg R. B. Ketzel M.. (2016). Road traffic and railway noise exposures and adiposity in adults: a cross-sectional analysis of the danish diet, cancer, and health cohort. Environ. Health Perspect. 124, 329–335. 10.1289/ehp.1409052 26241990 PMC4786981

[B10] Cross D. E. Halliwell B. Borish E. T. Pryor W. A. Ames B. A. Saul R. S.. (1987). Oxygen radicals and human disease. Ann. Intern. Med. 107, 526–545. 10.7326/0003-4819-107-4-526 3307585

[B11] Cui B. Wu M. She X. Liu H. (2012). Impulse noise exposure in rats causes cognitive deficits and changes in hippocampal neurotransmitter signaling and tau phosphorylation. Brain Res. 1427, 35–43. 10.1016/j.brainres.2011.08.035 22055774

[B12] Cui B. Wu M. Q. Zhu L. X. She X. J. Ma Q. Liu H. T. (2013). Effect of chronic noise exposure on expression of N-methyl-D-aspartic acid receptor 2B and Tau phosphorylation in hippocampus of rats. Biomed. Environ. Sci. 26, 163–168. 10.3967/0895-3988.2013.03.002 23425798

[B13] Emerit I. (1994). Reactive oxygen species, chromosome mutation, and cancer. Free Radic. Biol. Med. 16, 99–109. 10.1016/0891-5849(94)90246-1 8300000

[B15] Fornai F. Alessandrì M. G. Torracca M. T. Bassi L. Corsini G. U. (1997). Effects of noradrenergic lesions on MPTP/MPP^+^ kinetics and MPTP-induced nigrostriatal dopamine depletions. J. Pharmacol. Exp. Ther. 283, 100–107. 9336313

[B16] Fornai F. Bassi L. Torracca M. T. Alessandrì M. G. Scalori V. Corsini G. U. (1996). Region- and neurotransmitter-dependent species and strain differences in DSP-4-induced monoamine depletion in rodents. Neurodegeneration 5, 241–249. 10.1006/neur.1996.0032 8910902

[B17] Fornai F. Giorgi F. S. Alessandrì M. G. Giusiani M. Corsini G. U. (1999). Effects of pretreatment with *N*-(2-chloroethyl)-*N*-ethyl-2-bromobenzylamine (DSP-4) on methamphetamine pharmacokinetics and striatal dopamine losses. J. Neurochem. 72, 777–784. 10.1046/j.1471-4159.1999.0720777.x 9930753

[B18] Fornai F. Lenzi P. Frenzilli G. Gesi M. Ferrucci M. Lazzeri G.. (2004). DNA damage and ubiquitinated neuronal inclusions in the substantia nigra and striatum of mice following MDMA (ecstasy). Psychopharmacology 173, 353–363. 10.1007/s00213-003-1708-3 14673567

[B19] Fornai F. Ruffoli R. Giorgi F. S. Paparelli A. (2011). The role of locus coeruleus in the antiepileptic activity induced by vagus nerve stimulation. Eur. J. Neurosci. 33, 2169–2178. 10.1111/j.1460-9568.2011.07707.x 21535457

[B20] Frenzilli G. Lenzi P. Scarcelli V. Fornai F. Pellegrini A. Soldani P.. (2004). Effects of loud noise exposure on DNA integrity in rat adrenal gland. Environ. Health Perspect. 112, 1671–1672. 10.1289/ehp.7249 15579411 PMC1253657

[B21] Fuchs E. Czéh B. Kole M. H. Michaelis T. Lucassen P. J. (2004). Alterations of neuroplasticity in depression: the hippocampus and beyond. Eur. Neuropsychopharmacol. S481–S490. 10.1016/j.euroneuro.2004.09.002 15550346

[B22] Gáborján A. Lendvai B. Vizi E. S. (1999). Neurochemical evidence of dopamine release by lateral olivocochlear efferents and its presynaptic modulation in guinea-pig cochlea. Neuroscience 90, 131–138. 10.1016/s0306-4522(98)00461-8 10188940

[B23] Gale S. D. Person A. L. Perkel D. J. (2008). A novel basal ganglia pathway forms a loop linking a vocal learning circuit with its dopaminergic input. J. Comp. Neurol. 508, 824–839. 10.1002/cne.21700 18398824

[B24] George S. A. Knox D. Curtis A. L. Aldridge J. W. Valentino R. J. Liberzon I. (2013). Altered locus coeruleus-norepinephrine function following single prolonged stress. Eur. J. Neurosci. 37, 901–909. 10.1111/ejn.12095 23279008

[B202] Gesi M. Ferrucci M. Giusiani M. Lenzi P. Lazzeri G. Alessandrì M. G. (2004). Loud noise enhances nigrostriatal dopamine toxicity induced by MDMA in mice. Microsc. Res. Tech. 64, 297–303. 10.1002/jemt.20084 15481049

[B25] Gesi M. Santinami A. Ruffoli R. Conti G. Fornai F. (2001). Novel aspects of dopamine oxidative metabolism (confounding outcomes take place of certainties). Pharmacol. Toxicol. 89, 217–224. 10.1111/j.1600-0773.2001.890501.x 11881974

[B26] Goble T. J. Møller A. R. Thompson L. T. (2009). Acute high-intensity sound exposure alters responses of place cells in hippocampus. Hear. Res. 253, 52–59. 10.1016/j.heares.2009.03.002 19303432

[B27] Hartmann A. Agurell E. Beevers C. Brendler-Schwaab S. Burlinson B. Clay P.. (2003). Recommendations for conducting the *in vivo* alkaline Comet assay. 4th International Comet Assay Workshop. Mutagenesis 18, 45–51. 10.1093/mutage/18.1.45 12473734

[B28] Herbert H. Klepper A. Ostwald J. (1997). Afferent and efferent connections of the ventrolateral tegmental area in the rat. Anat. Embryol. (Berl) 196, 235–259. 10.1007/s004290050094 9310315

[B29] Hjortebjerg D. Andersen A. M. Christensen J. S. Ketzel M. Raaschou-Nielsen O. Sunyer J.. (2016). Exposure to road traffic noise and behavioral problems in 7-year-old children: a cohort study. Environ. Health Perspect. 124, 228–234. 10.1289/ehp.1409430 26126294 PMC4749080

[B30] Hours M. Bernard M. Arslan M. Montestrucq L. Richardson L. Deltour I.. (2009). Can loud noise cause acoustic neuroma? Analysis of the INTERPHONE study in France. Occup. Environ. Med. 66, 480–486. 10.1136/oem.2008.042101 19289391

[B32] Hu L. Zhao X. Yang J. Wang L. Yang Y. Song T.. (2014). Chronic scream sound exposure alters memory and monoamine levels in female rat brain. Physiol. Behav. 137, 53–59. 10.1016/j.physbeh.2014.06.012 24952268

[B33] Hudspeth A. J. (2000). “Hearing,” in Principles of Neural Science, 4th Edn. eds Kandel E. R. Schwartz J. H. Jessell T. M. (New York, NY: McGraw-Hill), 590–613.

[B34] Ising H. Braun C. (2000). Acute and chronic endocrine effects of noise: review of the research conducted at the Institute for Water, Soil and Air Hygiene. Noise Health 7, 7–24. 12689468

[B35] Kaltenbach J. A. (2006). The dorsal cochlear nucleus as a participant in the auditory, attentional and emotional components of tinnitus. Hear. Res. 216–217, 224–234. 10.1016/j.heares.2006.01.002 16469461

[B36] Kawecka-Jaszcz K. (1991). Effect of professional work and environmental factors on arterial blood pressure. Med. Pr. 42, 291–296. 1812390

[B37] Kim B. K. Ko I. G. Kim S. E. Kim C. J. Yoon J. S. Baik H. H.. (2013). Impact of several types of stresses on short-term memory and apoptosis in the hippocampus of rats. Int. Neurourol. J. 17, 114–120. 10.5213/inj.2013.17.3.114 24143289 PMC3797890

[B201] Klaude M. Eriksson S. Nygren J. Ahnström G. (1996). The comet assay: mechanisms and technical considerations. Mutat. Res. 363, 89–96. 10.1016/0921-8777(95)00063-1 8676929

[B38] Kromer L. F. Moore R. Y. (1980). Norepinephrine innervation of the cochlear nuclei by locus coeruleus neurons in the rat. Anat. Embryol. 158, 227–244. 10.1007/bf00315908 6986826

[B39] Kumaravel T. S. Jha A. N. (2006). Reliable Comet assay measurements for detecting DNA damage induced by ionising radiation and chemicals. Mutat. Res. 605, 7–16. 10.1016/j.mrgentox.2006.03.002 16621680

[B40] Lai S. W. Liao K. F. Lin C. L. Lin C. C. Sung F. C. (2014). Hearing loss may be a non-motor feature of Parkinson’s disease in older people in Taiwan. Eur. J. Neurol. 21, 752–757. 10.1111/ene.12378 24506292

[B41] Lang T. Fouriaud C. Jacquinet-Salord M. C. (1992). Length of occupational noise exposure and blood pressure. Int. Arch. Occup. Environ. Health 63, 369–372. 10.1007/bf00386929 1544682

[B42] Lemasters J. J. Caldwell-Kenkel J. C. Gao W. Nieminen A. L. Herman B. Thurman R. G. (1992). “Hypoxic, ischemic and reperfusion injury in the liver,” in Pathophysiology of Reperfusion Injury, ed. Das D. K. (Boca Raton, FL: CRC Press), 101–135.

[B43] Lenzi P. Frenzilli G. Gesi M. Ferrucci M. Lazzeri G. Fornai F.. (2003). DNA damage associated with ultrastructural alterations in rat myocardium after loud noise exposure. Environ. Health Perspect. 111, 467–471. 10.1289/ehp.5847 12676600 PMC1241429

[B44] Lovell D. P. Omori T. (2008). Statistical issues in the use of the comet assay. Mutagenesis 23, 171–182. 10.1093/mutage/gen015 18385511

[B45] Maison S. F. Liu X. P. Eatock R. A. Sibley D. R. Grandy D. K. Liberman M. C. (2012). Dopaminergic signaling in the cochlea: receptor expression patterns and deletion phenotypes. J. Neurosci. 32, 344–355. 10.1523/JNEUROSCI.4720-11.2012 22219295 PMC3313790

[B46] Manikandan S. Padma M. K. Srikumar R. Partasarathy N. J. Muthuvel A. Devi R. S. (2006). Effects of chronic noise stress on spatial memory of rats in relation to neuronal dendritic alteration and free radical-imbalance in hippocampus and medial prefrontal cortex. Neurosci. Lett. 399, 17–22. 10.1016/j.neulet.2006.01.037 16481110

[B47] Mulders W. H. Robertson D. (2001). Origin of the noradrenergic innervation of the superior olivary complex in the rat. J. Chem. Neuroanat. 21, 313–322. 10.1016/s0891-0618(01)00118-1 11429272

[B48] Mulders W. H. Robertson D. (2004). Dopaminergic olivocochlear neurons originate in the high frequency region of the lateral superior olive of guinea pigs. Hear. Res. 187, 122–130. 10.1016/s0378-5955(03)00308-3 14698093

[B49] Mulders W. H. Robertson D. (2005). Catecholaminergic innervation of guinea pig superior olivary complex. J. Chem. Neuroanat. 30, 230–242. 10.1016/j.jchemneu.2005.09.005 16236480

[B50] Niu X. Tahera Y. Canlon B. (2007). Environmental enrichment to sound activates dopaminergic pathways in the auditory system. Physiol. Behav. 92, 34–39. 10.1016/j.physbeh.2007.05.020 17631367

[B51] Ohlemiller K. K. Wright J. S. Dugan L. L. (1999). Early elevation of cochlear reactive oxygen species following noise exposure. Audiol. Neurootol. 4, 229–236. 10.1159/000013846 10436315

[B52] Okada A. Ariizumi M. Okamoto G. (1983). Changes in cerebral norepinephrine induced by vibration or noise stress. Eur. J. Appl. Physiol. Occup. Physiol. 52, 94–97. 10.1007/bf00429032 6686136

[B53] Orban E. McDonald K. Sutcliffe R. Hoffmann B. Fuks K. B. Dragano N.. (2016). Residential road traffic noise and high depressive symptoms after five years of follow-up: results from the heinz nixdorf recall study. Environ. Health Perspect. 124, 578–585. 10.1289/ehp.1409400 26606640 PMC4858388

[B54] Paxinos G. Watson C. (1986). The Rat Brain in Stereotaxic Coordinates. 2nd Edn. San Diego, CA: Academic Press.

[B56] Pisani V. Sisto R. Moleti A. Di Mauro R. Pisani A. Brusa L.. (2015). An investigation of hearing impairment in *de-novo* Parkinson’s disease patients: a preliminary study. Parkinsonism Relat. Disord. 21, 987–991. 10.1016/j.parkreldis.2015.06.007 26071125

[B57] Preston-Martin S. Thomas D. C. Wright W. E. Henderson B. E. (1989). Noise trauma in the aetiology of acoustic neuromas in men in Los Angeles County. 1978–1985. Br. J. Cancer. 59, 783–786. 10.1038/bjc.1989.163 2736213 PMC2247243

[B58] Rabat A. (2007). Extra-auditory effects of noise in laboratory animals: the relationship between noise and sleep. J. Am. Assoc. Lab. Anim. Sci. 46, 35–41. 17203914

[B59] Ramos B. P. Arnsten A. F. (2007). Adrenergic pharmacology and cognition: focus on the prefrontal cortex. Pharmacol. Ther. 113, 523–536. 10.1016/j.pharmthera.2006.11.006 17303246 PMC2151919

[B60] Ravindran R. Rathinasamy S. D. Samson J. Senthilvelan M. (2005). Noise-stress-induced brain neurotransmitter changes and the effect of Ocimum sanctum (Linn) treatment in albino rats. J. Pharmacol. Sci. 98, 354–360. 10.1254/jphs.fp0050127 16113498

[B61] Ruffoli R. Giorgi F. S. Pizzanelli C. Murri L. Paparelli A. Fornai F. (2011). The chemical neuroanatomy of vagus nerve stimulation. J. Chem. Neuroanat. 42, 288–296. 10.1016/j.jchemneu.2010.12.002 21167932

[B62] Säljö A. Bao F. Jingshan S. Hamberger A. Hansson H. A. Haglid K. G. (2002). Exposure to short-lasting impulse noise causes neuronal c-Jun expression and induction of apoptosis in the adult rat brain. J. Neurotrauma 19, 985–991. 10.1089/089771502320317131 12225658

[B63] Samson J. Sheeladevi R. Ravindran R. Senthilvelan M. (2007). Stress response in rat brain after different durations of noise exposure. Neurosci. Res. 57, 143–147. 10.1016/j.neures.2006.09.019 17092591

[B64] Sedelis M. Hofele K. Auburger G. W. Morgan S. Huston J. P. Schwarting R. K. (2000). MPTP susceptibility in the mouse: behavioral, neurochemical, and histological analysis of gender and strain differences. Behav. Genet. 30, 171–182. 11105391 10.1023/a:1001958023096

[B65] Sikandaner H. E. Park S. Y. Kim M. J. Park S. N. Yang D. W. (2017). Neuroprotective effects of sildenafil against oxidative stress and memory dysfunction in mice exposed to noise stress. Behav. Brain Res. 319, 37–47. 10.1016/j.bbr.2016.10.046 27836585

[B66] Singh N. P. (2000). A simple method for accurate estimation of apoptotic cells. Exp. Cell Res. 256, 328–337. 10.1006/excr.2000.4810 10739681

[B200] Singh N. P. Lai H. Khan A. (1995). Ethanol-induced single-strand DNA breaks in rat brain cells. Mutat. Res. 345, 191–196. 10.1016/0165-1218(95)90054-3 8552140

[B67] Singh N. P. McCoy M. T. Tice R. R. Schneider E. L. (1988). A simple technique for quantitation of low levels of DNA damage in individual cells. Exp. Cell Res. 175, 184–191. 10.1016/0014-4827(88)90265-0 3345800

[B68] Tanaka M. Yoshida M. Emoto H. Ishii H. (2000). Noradrenaline systems in the hypothalamus, amygdala and locus coeruleus are involved in the provocation of anxiety: basic studies. Eur. J. Pharmacol. 405, 397–406. 10.1016/s0014-2999(00)00569-0 11033344

[B69] Tice R. R. Agurell E. Anderson D. Burlinson B. Hartmann A. Kobayashi H.. (2000). Single cell gel/comet assay: guidelines for *in vitro* and *in vivo* genetic toxicology testing. Environ. Mol. Mutagen. 35, 206–221. 10.1002/(SICI)1098-2280(2000)35:3<206::AID-EM8>.0.CO;2-J 10737956

[B70] Tsai H. Y. Lu Y. H. Wu C. R. Chen Y. F. (2005). Effects of noise on monoamine levels in the rat brain using *in vivo* microdialysis. Pflugers Arch. 450, 83–87. 10.1007/s00424-004-1372-4 15614574

[B71] Uran S. L. Caceres L. G. Guelman L. R. (2010). Effects of loud noise on hippocampal and cerebellar-related behaviours. Role of oxidative state. Brain Res. 1361, 102–114. 10.1016/j.brainres.2010.09.022 20846514

[B72] Van Campen L. E. Murphy W. J. Franks J. R. Mathias P. I. Toraason M. A. (2002). Oxidative DNA damage is associated with intense noise exposure in the rat. Hear. Res. 164, 29–38. 10.1016/s0378-5955(01)00391-4 11950522

[B73] Van de Kar L. D. Blair M. L. (1999). Forebrain pathways mediating stress-induced hormone secretion. Front. Neuroendocrinol. 20, 1–48. 10.1006/frne.1998.0172 9882535

[B74] Vicente-Torres M. A. Gil-Loyzaga P. (1999). Noise stimulation decreases the concentration of norepinephrine in the rat cochlea. Neurosci. Lett. 266, 217–219. 10.1016/s0304-3940(99)00305-5 10465712

[B75] Vitale C. Marcelli V. Allocca R. Santangelo G. Riccardi P. Erro R.. (2012). Hearing impairment in Parkinson’s disease: expanding the nonmotor phenotype. Mov. Disord. 27, 1530–1535. 10.1002/mds.25149 23032708

[B76] WHO (World Health Organization) (2011). Burden of disease from environmental noise: quantification of healthy life years lost in europe, bonn, germany: WHO, regional office for europe, european centre for environmental and health. Available online at: http://www.euro.who.int/_data/assets/pdf_file/0008/136466/e94888.pdf

[B77] Yeomans J. S. Frankland P. W. (1995). The acoustic startle reflex: neurons and connections. Brain Res. Rev. 21, 301–314. 10.1016/0165-0173(96)00004-5 8806018

[B78] Yurek D. M. Deutch A. Y. Roth R. H. Sladek J. R. Jr. (1989). Morphological, neurochemical and behavioral characterizations associated with the combined treatment of diethyldithiocarbamate and 1-methyl-4-phenyl-1,2,3,6-tetrahydropyridine in mice. Brain Res. 497, 250–259. 10.1016/0006-8993(89)90270-9 2573405

